# Subcentimeter Pulmonary Nodules Detected in Patients with Sarcoma

**DOI:** 10.1080/13577140020008110

**Published:** 2000-09

**Authors:** M. S. Ginsberg, D. M. Panicek


					Sarcoma (2000) 4, 135
ERRATUM
Subcentimeter pulmonary nodules detected in patients with sarcoma
M. S. GINSBERG & D. M. PANICEK
We apologise for the poor quality of reproduction of
the original (R) gure.(SubcentimeterPulmonaryNodules
Detected in Patients with Sarcoma, Ginsberg and
Panicek, Figure 1, Sarcoma, Volume 4, Issue 1 2, pp.
63 66). Below please (R) nd the correct version which
demonstrates the lesion as the authors intended.
Figure 1. An 18-year-old male with high grade synovial sarcoma of arm diagnosed 3 months earlier. Helical CT shows 0.8 3 0.6 cm
left lower lobe lung nodule (arrow) not visible on prior helical CT. Histopathological examination of wedge resection specimen
demonstrated a benign intrapulmonary lymph node.
1357-714Xprint/1369-1643online/00/030135-01 1/2 2000Taylor & Francis Ltd
DOI: 10.1080/13577140020008110

				

